# Efficacy and Safety of the Reduced Bivalirudin in Patients Undergoing Coronary Angiography or Percutaneous Coronary Intervention Stratified by Renal Function (REDUCE BOLUS): A Single-Blind, Stratified Randomized, Non-inferiority Trial

**DOI:** 10.3389/fcvm.2022.864048

**Published:** 2022-04-25

**Authors:** Qiang Hu, Ya-Ling Han, Tie-Nan Zhou, Xiao-Zeng Wang, Quan-Yu Zhang

**Affiliations:** ^1^Department of Cardiology, General Hospital of Northern Theater Command, Shenyang, China; ^2^Department of Cardiology, Xijing Hospital, Air Force Medical University, Xi’an, China

**Keywords:** bivalirudin, loading dose, percutaneous coronary intervention, bleeding, activated clotting time

## Abstract

**Background:**

In clinical practice, some cases indicated that the loading dose of bivalirudin increased the bleeding risk, particularly in patients with renal insufficiency. Therefore, this study aimed to assess the efficacy and safety of the low-dose (80%) bolus injection of bivalirudin in patients undergoing cardiac catheterization stratified by renal function.

**Methods:**

A total of 204 individuals in the REDUCE BOLUS trial were stratified 1:1 to the estimated glomerular filtration rate (eGFR) ≥ 60 ml/min cohort or eGFR < 60 ml/min cohort, then randomized 1:1 to the reduced bolus bivalirudin group (i.e., the experimental group) or normal bolus bivalirudin group (i.e., the control group), respectively. The primary end point was to compare the differences of the area under the curve of activated clotting time (ACT) between the two groups. The secondary end points were the postoperative net adverse clinical events (NACEs) before discharge, defined as the all-cause mortality, recurrent myocardial infarction, ischemia-driven target vessel revascularization, stroke, and bleeding events.

**Results:**

Between January 3, 2020, and March 26, 2021, 204 patients undergoing coronary angiography were randomly assigned, including 102 (i.e., 51 in the control group and 51 in the experimental group) with normal eGFR and 102 (i.e., 51 control and 51 experimental) with abnormal eGFR. No difference was observed in the curve of ACT between the control group and the experimental group (0.55 ± 0.09 vs. 0.56 ± 0.08, *P* = 0.542 and 0.55 ± 0.06 vs. 0.57 ± 0.05, *P* = 0.075, respectively, for normal eGFR cohort and abnormal eGFR cohort). The one-sided 97.5% lower confidence bound for the difference in the area under the ACT curve was –0.017 and 0.0015 in eGFR ≥ 60 ml/min and eGFR<60 ml/min cohort, respectively, both above the preset non-inferiority criterion of -0.07, establishing the non-inferiority. There was no incidence of NACE and stent thrombosis before discharge in each group.

**Conclusion:**

In patients undergoing cardiac catheterization, the efficacy and safety of the reduced bolus of bivalirudin were non-inferior to the normal one, even in patients without chronic kidney disease.

**Clinical Trial Registration:**

[www.ClinicalTrials.gov], identifier [NCT03588611].

## Introduction

Coronary heart disease (CHD) is one of the common cardiovascular diseases that threatens people’s health around the world (1). Percutaneous coronary intervention (PCI), which has been demonstrated to be the optimum reperfusion strategy for patients presenting with ST-segment elevation infarction (STEMI) (2), requires the optimum regime for adjunctive antithrombotic treatment with antiplatelet and antithrombotic drugs. The two most commonly used antithrombotic drugs worldwide are unfractionated heparin (UFH) and bivalirudin. Bivalirudin, a direct thrombin inhibitor, is able to prolong the dose-dependent activated partial thromboplastin time (aPTT), thrombin time (TT), and prothrombin time (PT) by inhibiting thrombin directly and reversibly without affecting the platelet function and causing heparin-induced thrombocytopenia. It also has a predictable anticoagulation effect and does not need any antithrombin compared with UFH (3). Meanwhile, it may be superior to UFH for its shorter half-life, less immunogenicity, and fewer side effects, especially fewer bleeding complications (3). Numerous clinical trials have shown that the bivalirudin reduced the risk of bleeding in patients with CHD compared with heparin or heparin plus glycoprotein IIb/IIIa inhibitors (GPIs) (4–8), a finding sustained at 3 years (9). However, two previous trials have shown that the bleeding risk was not lower in patients receiving bivalirudin than those receiving heparin (10, 11). Moreover, the increase in the bleeding risk was associated with concomitant therapy with UFH, warfarin, and any other antithrombotic agents or even with the low body weight (12). Consequently, the less guidance-recommended doses of bivalirudin were needed, especially for people in Asia with a diminutive stature.

Chronic kidney disease (CKD) is highly related to cardiovascular disease. The incidence of CKD in patients with STEMI and non-STEMI (NSTEMI) is 30.5 and 42.9%, respectively (13). CKD predominantly affects the prognosis of patients with CHD, which may increase ischemic and bleeding events (14–17). In addition, the usage of anticoagulants, especially those that are excreted by the kidney, may also increase the bleeding risk for patients with poor renal function (16, 18). A previous study has demonstrated that the clearance rate of bivalirudin was 4.8 ml/min/kg for patients with estimated glomerular filtration rate (eGFR) ≥ 60 ml/min, while the clearance rate was 2.5 ml/min/kg or less in patients with eGFR <60 ml/min (19). As a result, bivalirudin may be used in such patients with dosage modification, although it is safer than heparin either with GPIs or without GPIs (14, 20). However, most previous studies assessing the bleeding risk of anticoagulants have excluded patients with CKD (21). Accordingly, the treatment strategy of bivalirudin in patients with CHD with coherent CKD remains unclear.

To investigate these hypothesized benefits, REDUCE BOLUS trial, a single-center, single-blind, stratified randomized trial, was performed to test whether the reduced-bolus injection of bivalirudin in patients undergoing coronary angiography or PCI with or without renal insufficiency was non-inferior to the normal-bolus injection of bivalirudin.

## Materials and Methods

### Trial Overview

REDUCE BOLUS is a prospective, single-center, single-blind, stratified randomized trial designed to evaluate the efficacy and safety of low-bolus injection of bivalirudin (0.6 mg/kg) in patients undergoing coronary angiography or PCI with or without renal insufficiency.

### Enrollment Criteria

The eligible criteria for the study included patients aged 18 years or older, requiring coronary angiography or PCI after the medical judgments, agreeing to use bivalirudin during the operation, and willing to provide the informed consent. Major exclusion criteria included patients on dialysis; pregnancy or lactation; allergy to any of the study drugs or devices; any condition that makes coronary angiography or PCI unsuitable, e.g., cardiogenic shock; thrombolytic therapy within 72 h of the onset of STEMI; thrombolytic therapy administered before randomization or any anticoagulant administered within 48 h of randomization; active or recent major bleeding or bleeding predisposition including recent hemorrhage in the retina or vitreous within 1 month, hemorrhage in the gastrointestinal tract or the urinary tract within 3 months, cerebral hemorrhage within 6 months or cerebral infarction within 3 months; secondary hemorrhage caused by other diseases, such as active gastric ulcer and active ulcerative colitis; major surgery within 1 month; clinical syndrome suspicious for aortic dissection, pericarditis, or endocarditis; blood pressure higher than 180/110 mmHg; hemoglobin less than 100 g/dl; platelet count less than 100 × 10^9^/L; aminotransferase level greater than 3 × the upper limit of normal; patients with progressive severe diseases such as cancer or diseases that make patients exhausted and anticipated to live no longer than 1 year; patients who are considered by the researchers to be unsuitable for study participation; and patient unwilling or unable to provide written informed consent. The study protocol was approved by the Institutional Ethics Committee of the General Hospital of Northern Theater Command and was conducted in accordance with the Declaration of Helsinki. All patients provided the informed consent before randomization.

### Randomization and Treatment

**FIGURE 1:**
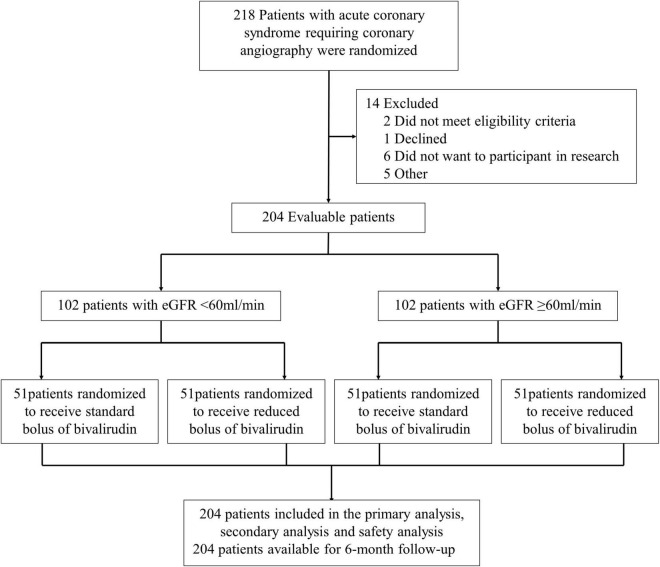
Flow diagram of the study.

The participants who met all entry criteria were eligible to participate in our clinical trial. As shown in Figure 1, all the participants undergoing coronary angiography or PCI would be stratified 1:1 to the abnormal eGFR cohort (eGFR < 60 ml/min) and normal eGFR cohort (eGFR ≥ 60 ml/min). Then, the participants in each group would be randomly assigned to the intervention group (low-dose bolus injection of bivalirudin, 0.6 mg/kg) or control group (normal-dose bolus injection of bivalirudin, 0.75 mg/kg) in a 1:1 ratio using sealed envelopes. In the intervention group, the participants received the low-dose bolus injection of bivalirudin (Salubris Pharmaceuticals Co.) before coronary angiography, which accounted for around 80% of the standard dose. In contrast, in the control group, the participants used the normal-dose bolus injection of bivalirudin. In addition, all the participants received the maintenance dose of bivalirudin at the speed of 1.75 mg/kg/h during the PCI procedure and for at least 30 min but no more than 4 h afterward, which are adjusted by the activated clotting time (ACT) immediately after the initial bolus of bivalirudin. The ACT would be measured at the baseline and 5, 10, and 30 min after administering bivalirudin using the ACT plus (Medtronic Public Limited Company). If the ACT measured at 5 min after the initial bolus was less than 225 s, the participants would receive an additional bolus injection of about 0.3 mg/kg. Provisional tirofiban was administrated in each group for no-reflow or other thrombotic complications.

The baseline information and the results of blood tests, urine routine, and other conventional tests were collected via the cv-net clinical data collection system (Beijing Crealife Technology Co., Ltd.). Moreover, the net adverse clinical events (NACEs), and the safety end points were assessed by the cardiologists who were not aware of the treatments received in the whole process of the study. Guidance-recommended medical management and the same approach would be implemented to each group according to the contemporary guidelines (22).

### End Points and Definitions

**FIGURE 2:**
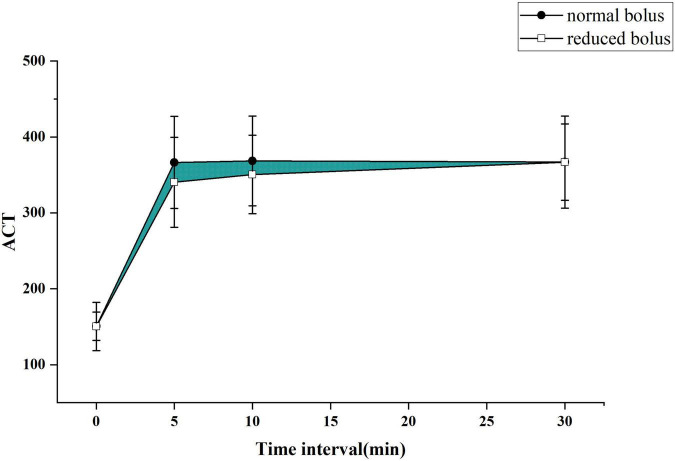
The activated clotting time (ACT) curve in each group for patients with normal estimated glomerular filtration rate (eGFR).

**FIGURE 3:**
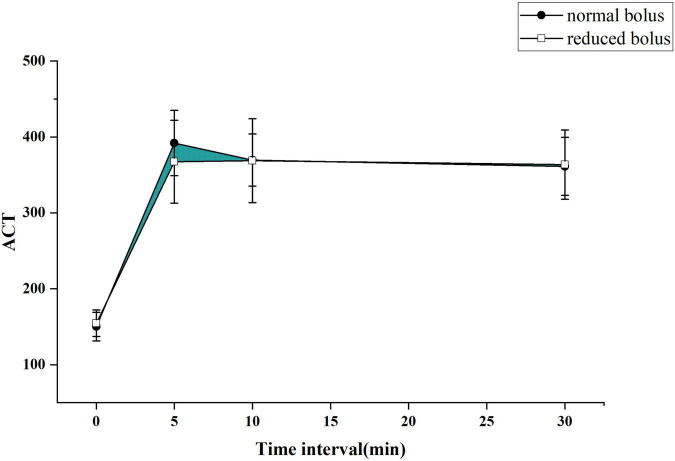
The ACT curve in each group for patients with abnormal eGFR.


The primary end point was to compare the differences of the area under the curve of ACT between two groups as depicted in Figures 2, 3 for both the eGFR ≥ 60 ml/min and eGFR < 60 ml/min cohorts. The cardiologist measured the ACT at the baseline and at 5, 10, and 30 min after administering bivalirudin. Therefore, we built two ACT curves by connecting the four time points in order so that we got images, as shown in Figures 2, 3. The images indicated the area under the curve of ACT was different between the two groups where the normal bolus was higher than the reduced one. The shaded part in the figure, defined as the difference between the areas of two curves, would represent the efficacy difference between the two groups. The area under the curve of ACT was calculated using the trapezoidal method. To broadly depict the trend of change in ACT, we utilized the relative area under the curve of ACT, calculated as the ratio of absolute differences of the curve and the whole area under the curve. The formula for the calculation is as follows.

The⁢relative⁢area⁢under⁢curve

=The⁢area⁢under⁢the⁢curve-ACT⁢value⁢at⁢ 0⁢min*30The⁢area⁢under⁢the⁢curve

Major secondary end points were the rate of postoperative NACEs before hospital discharge. The NACE was defined as a composite of major adverse cardiac or cerebral events (MACCEs) (e.g., all-cause mortality, recurrent myocardial infarction, ischemia-driven target vessel revascularization, stroke) or any bleeding defined by the Bleeding Academic Research Consortium (BARC) definition (23). The key safety end point of the study included the incidence of in-stent thrombosis and the bleeding events for the duration of the hospital after the operation, which was defined according to the Academic Research Consortium (ARC) (24) and the BARC, respectively.

A clinical follow-up was performed 6 months after hospital discharge. All net adverse events and stent thrombosis were collected. The clinical events would be adjudicated by an independent clinical events committee, which was not aware of any information regarding the allocation.

### Sample Size and Statistical Analysis

Based on the result of our preliminary experiment, the area under the curve of ACT in the normal-bolus group was 0.56 ± 0.099, while the area in the reduced-bolus group was 0.53 ± 0.05. A total of 204 evaluable patients were required to test for a non-inferiority margin of –0.07, with 90% power. Consequently, there would be 51 subjects in each group. The non-inferiority margin was determined in accordance with clinical relevance and experience—we regarded the reduced bolus of bivalirudin as clinically non-inferior if the area of ACT in the reduced-bolus group decreased by no more than 0.07.

Continuous variables were expressed as mean ± *SD* and were compared between the groups using the independent *t*-test if they were normally distributed. But if they were not normally distributed, the standard transformation would be applied to achieve the approximately normal distribution. In addition, they would be expressed as median and compared using the nonparametric test. Categorical variables were expressed as numbers (percent) and were compared using the chi-square test or Fisher’s exact test. We did the 6-month event analyses of NACEs and any bleeding with time-to-event data (for which patients were censored at the time of withdrawal from the study or at last follow-up), which were displayed with Kaplan-Meier plots and were compared with the log-rank test. Consistency of treatment effect in reduced-bolus group for the NACE and any bleeding compared with the normal bolus of bivalirudin was examined in 12 prespecified subgroups. The non-inferiority test with a 1-sided 97.5%CI was used to test the non-inferiority of reduced bolus compared with the normal bolus. Except for the non-inferiority test, all other tests were 2-tailed. A *P*-value < 0.05 is considered significant for superiority. All statistical analyses were performed using SPSS version 24.0.

## Results

### Patients

A total of 204 patients with the acute coronary syndrome (ACS) undergoing coronary angiography were enrolled in the trial from January 3, 2020, through March 26, 2021 (Figure 1). All the participants undergoing coronary angiography or PCI would be stratified 1:1 to the abnormal eGFR and normal eGFR cohorts. Then, 102 participants in each group would be randomly assigned 1:1 to the reduced-bolus group (*n* = 51) or normal-bolus group (*n* = 51). The baseline characteristics of patients were comparable between the two groups (Tables 1, 2). The whole data set of participants [147 (72.1% male)] had a mean (*SD*) age of 65.86 (10.58) years, and most had unstable angina [122 (59.8%)].

**TABLE 1:** Baseline characteristics according to the randomized treatment.

Characteristics	eGFR ≥ 60 ml/min			eGFR < 60 ml/min		
	Reduced-bolus (*n* = 51)	Normal-bolus (*n* = 51)	*P*-value	Reduced-bolus (*n* = 51)	Normal-bolus (*n* = 51)	*P*-value
Age (years)	61.76 ± 10.63	61.96 ± 9.36	0.921	69.59 ± 8.66	70.12 ± 10.67	0.784
>65 years, no. (%)	18 (35.3%)	16 (31.4%)	0.674	39 (76.5%)	39 (76.5%)	1.000
Men, no. (%)	40 (78.4%)	38 (74.5%)	0.641	33 (64.7%)	36 (70.6%)	0.525
Weight (kg)	72.34 ± 10.66	71.56 ± 9.81	0.700	70.64 ± 9.95	69.63 ± 12.10	0.646
Body-mass index (kg/m2)*^a^*	25.34 ± 2.98	25.04 ± 2.82	0.605	25.03 ± 3.01	24.76 ± 3.69	0.692
**Diabetes, no. (%)**	19 (37.3%)	14 (27.5%)	0.290	28 (54.9%)	28 (54.9%)	1.000
Insulin-dependent	6 (11.8%)	4 (7.8%)	0.505	19 (37.3%)	16 (31.4%)	0.532
Non-insulin-dependent	13 (25.5%)	10 (19.6%)	0.477	9 (17.6%)	12 (23.5%)	0.463
**Smoking status, no. (%)**						
Current smoker	18 (35.3%)	22 (43.1%)	0.417	10 (19.6%)	5 (9.8%)	0.162
Previous smoker	11 (21.6%)	8 (15.7%)	0.445	13 (25.5%)	16 (31.4%)	0.510
Hyperlipidemia, no. (%)	18 (35.3%)	16 (31.4%)	0.674	23 (45.1%)	27 (52.9%)	0.428
Hypertension, no. (%)	36 (70.6%)	34 (66.7%)	0.670	42 (82.4%)	43 (84.3%)	0.790
**Previous myocardial infarction, no. (%)**	15 (29.4%)	12 (23.5%)	0.501	15 (29.4%)	17 (33.3%)	0.670
STEMI	13 (25.5%)	11 (21.6%)	0.641	11 (21.6%)	12 (23.5%)	0.813
NSTEMI	2 (3.9%)	1 (2.0%)	1.000	4 (7.8%)	5 (9.8%)	1.000
Previous PCI, no. (%)	12 (23.5%)	15 (29.4%)	0.501	15 (29.4%)	20 (39.2%)	0.297
Previous CABG, no. (%)	2 (3.9%)	0 (0.0%)	0.475	4 (7.8%)	2 (3.9%)	0.674
Previous TIA or stroke, no. (%)	13 (25.5%)	7 (13.7%)	0.135	17 (33.3%)	13 (25.5%)	0.385
Peripheral vascular disease, no. (%)	3 (5.9%)	2 (3.9%)	1.000	6 (11.8%)	2 (3.9%)	0.269
Chronic obstructive pulmonary disease, no. (%)	0 (0.0%)	0 (0.0%)	1.000	0 (0%)	2 (3.9%)	0.475
Chronic kidney disease, no. (%)	1 (2.0%)	0 (0.0%)	1.000	15 (29.4%)	13 (25.5%)	0.657
Dialysis, no. (%)	0 (0.0%)	0 (0.0%)	1.000	2 (3.9%)	0 (0%)	0.475
Anemia, no. (%)*^b^*	9 (17.6%)	10 (19.6%)	0.799	30 (58.8%)	26 (51%)	0.426

*STEMI, ST-segment elevation myocardial infarction; NSTEMI, non-ST-segment elevation myocardial infarction; PCI, percutaneous coronary intervention; CABG, coronary artery bypass grafting; TIAs, transient ischemic attacks.*

*^a^Body mass index was calculated as weight in kilograms divided by height in meters squared.*

*^b^Anemia was determined by hemoglobin less than 13 g/dl for men or less than 12 g/dl for women.*

**TABLE 2:** Clinical presentation and medications at baseline.

Characteristics	eGFR ≥ 60 ml/min			eGFR < 60 ml/min		
	Reduced-bolus (*n* = 51)	Normal-bolus (*n* = 51)	*P*-value	Reduced-bolus (*n* = 51)	Normal-bolus (*n* = 51)	*P*-value
STEMI, no. (%)	9 (17.6%)	9 (17.6%)	1.000	7 (13.7%)	8 (15.7%)	0.780
**NSTE-ACS, no. (%)**	42 (82.4%)	42 (82.4%)	1.000	44 (86.3%)	43 (84.3%)	0.780
Troponin-negative	29 (56.9%)	33 (64.7%)	0.417	32 (62.7%)	37 (72.5%)	0.290
Troponin-positive	13 (25.5%)	9 (17.6%)	0.336	12 (23.55%)	6 (11.8%)	0.119
ST-segment deviation	13 (25.5%)	9 (17.6%)	0.336	14 (27.5%)	17 (33.3%)	0.518
T-wave inversion	20 (39.2%)	17 (33.3%)	0.537	23 (45.1%)	18 (35.3%)	0.313
**Symptom onset to hospital arrival (h)**	96 (24–360)	168 (24–480)	0.454	240 (72–720)	360 (168–720)	0.231
STEMI	24 (6.5–84)	11 (6.75–56.5)	0.478	16 (6–192)	72 (48–240)	0.221
NSTE-ACS	168 (42–540)	204 (90–720)	0.250	288 (84–1,260)	720 (168–1,440)	0.251
Killip class II, III, or IV, no. (%)	3 (5.9%)	3 (5.9%)	1.000	7 (13.7%)	7 (13.7%)	1.000
Previous lytic therapy, no. (%)	1 (2.0%)	1 (2.0%)	1.000	1 (2.0%)	1 (2.0%)	1.000
Systolic arterial pressure (mmHg)	136.16 ± 17.28	133.33 ± 22.03	0.473	138.45 ± 22.38	138.14 ± 19.96	0.941
Heart rate (beats/min)	78.02 ± 12.79	76.18 ± 10.97	0.437	78.37 ± 13.12	78.25 ± 11.09	0.961
Cardiopulmonary resuscitation before arrival at the catheterization laboratory, no. (%)	0 (0.0%)	2 (3.9%)	0.475	3 (5.9%)	0 (0.0%)	0.241
eGFR (ml/min/1.73 m^2^)	99.73 ± 25.36	100.98 ± 20.92	0.785	48.97 ± 15.00	47.99 ± 10.80	0.706
**CRUSADE bleeding score *^a^***	21.76 ± 12.17	21.45 ± 9.05	0.883	43.43 ± 11.41	42.16 ± 9.21	0.536
> 30 (moderate or high bleeding risk), no. (%)	11 (21.6%)	7 (13.7%)	0.299	45 (88.2%)	45 (88.2%)	1.000
**Medications during the hospitalization, no. (%)**						
Aspirin	51 (100%)	51 (100%)	1.000	51 (100%)	48 (94.1%)	0.241
Clopidogrel	41 (80.4%)	41 (80.4%)	1.000	42 (82.4%)	46 (90.2%)	0.250
Ticagrelor	15 (29.4%)	16 (31.4%)	0.830	18 (35.3%)	10 (19.6%)	0.076
Fondaparinux	1 (2.0%)	0 (0.0%)	1.000	3 (5.9%)	7 (13.7%)	0.183
Angiotensin-converting enzyme inhibitors	15 (29.4%)	20 (39.2%)	0.297	8 (15.7%)	15 (29.4%)	0.097
Angiotensin II receptor blockers	17 (33.3%)	11 (21.6%)	0.183	34 (66.7%)	32 (62.7%)	0.679
Statins	51 (100%)	49 (96.1%)	0.475	50 (98.0%)	51 (100%)	1.000
Beta-blockers	35 (68.6%)	32 (62.7%)	0.532	43 (84.3%)	37 (72.5%)	0.149
Calcium channel blocker	17 (33.3%)	16 (31.4%)	0.832	32 (62.7%)	24 (47.1%)	0.111
Diuretic	14 (27.5%)	20 (39.2%)	0.208	30 (58.8%)	23 (45.1%)	0.165
Proton-pump inhibitor	45 (88.2%)	47 (92.2%)	0.505	46 (90.2%)	45 (88.2%)	0.750
Unfractionated heparin	2 (3.9%)	2 (3.9%)	1.000	6 (11.8%)	5 (9.8%)	0.750
Low molecular weight heparin	5 (9.8%)	5 (9.8%)	1.000	8 (15.7%)	5 (9.8%)	0.373
Enoxaparin	34 (66.7%)	30 (58.8%)	0.413	16 (31.4%)	18 (35.3%)	0.674
Rivaroxaban	0 (0.0%)	0 (0.0%)	1.000	3 (5.9%)	3 (5.9%)	1.000
Bivalirudin	51 (100%)	51 (100%)	1.000	51 (100%)	51 (100%)	1.000
Glycoprotein IIb/IIIa inhibitor	6 (11.8%)	10 (19.6%)	0.276	5 (9.8%)	4 (7.8%)	1.000

*STEMI, ST-segment elevation myocardial infarction; NSTE-ACS, Non-ST-elevation acute coronary syndrome; eGFR, estimated glomerular filtration rate; CRUSADE, Can Rapid Risk Stratification of Unstable Angina Patients Suppress Adverse Outcomes With Early Implementation of the ACC/AHA Guidelines.*

*^a^CRUSADE bleeding score denotes the risk of bleeding with higher numbers representing a greater risk of bleeding.*

### Treatment and Procedure

Characteristics of the patients are summarized in Table 3, and characteristics of the lesions are summarized in Table 4. All the patients received the bivalirudin in the catheterization laboratory. The dose was lower in the reduced-bolus group than that in the normal-bolus group for both eGFR cohorts (9.50 ± 1.45 vs. 10.81 ± 1.55, *P* = 0.000; 9.61 ± 1.54 vs. 10.30 ± 1.76, *P* = 0.037, respectively). The maintained dose of bivalirudin was comparable between the two groups for both eGFR cohorts (23.44 ± 3.62 vs. 25.03 ± 3.37, *P* = 0.023; 22.71 ± 3.53 vs. 23.40 ± 4.64, *P* = 0.396, respectively). Provisional GPIs were used in 6 (5.9%) patients in the reduced-bolus group and 7 (7.8%) patients in the normal-bolus group. The average maximum ACT during the operation between the reduced-bolus group and normal-bolus group was 385.61 ± 53.24 s and 402.57 ± 61.90 s for the normal eGFR cohort, respectively (*P* = 0.141), and for the abnormal eGFR cohort, 387.08 ± 56.80 s and 400.84 ± 40.20 s, respectively (*P* = 0.161). The postoperative administration of bivalirudin, enoxaparin, and low-molecular-weight heparin before hospital discharge between the two groups for both cohorts was comparable (*P* > 0.05).

**TABLE 3:** Angiographic patient-based procedural characteristics.

Characteristics	eGFR ≥ 60 ml/min			eGFR < 60 ml/min		
	Reduced-bolus (*n* = 51)	Normal-bolus (*n* = 51)	*P*-value	Reduced-bolus (*n* = 51)	Normal-bolus (*n* = 51)	*P*-value
**Medications in the catheterization laboratory**						
Bivalirudin, no. (%)	51 (100.0%)	51 (100.0%)	1.000	51 (100%)	51 (100%)	1.000
Loading dose (ml)	9.50 ± 1.45	10.81 ± 1.55	0.000	9.61 ± 1.54	10.30 ± 1.76	0.037
Maintenance dose (ml/h)	23.44 ± 3.62	25.03 ± 3.37	0.023	22.71 ± 3.53	23.40 ± 4.64	0.396
Unfractionated heparin, no. (%)	2 (3.9%)	2 (3.9%)	1.000	6 (11.8%)	5 (9.8%)	0.750
Tirofiban, no. (%)	3 (5.9%)	5 (9.8%)	0.713	3 (5.9%)	3 (5.9%)	1.000
Additional bolus of bivalirudin, no. (%)	0 (0.0%)	0 (0.0%)	1.000	0 (0.0%)	0 (0.0%)	1.000
Contrast agent (ml)	120 (110–210)	140 (80–210)	0.655	120 (90–220)	120 (60–150)	0.232
Intra-aortic balloon pump, no. (%)	0 (0.0%)	2 (3.9%)	0.475	1 (2.0%)	1 (2.0%)	1.000
**Arterial access site, no. (%)**						
Femoral	0 (0.0%)	1 (2.0%)	1.000	3 (5.9%)	5 (9.8%)	0.713
Radial	50 (98.0%)	50 (98.0%)	1.000	43 (84.3%)	45 (88.2%)	0.565
Both	1 (2.0%)	0 (0.0%)	1.000	5 (9.8%)	1 (2.0%)	0.207
**Number of diseased coronary vessels, no. (%)**						
1	3 (5.9%)	5 (9.8%)	0.905	9 (17.6%)	5 (9.8%)	0.180
2	14 (27.5%)	14 (27.5%)		15 (29.4%)	10 (19.6%)	
3	33 (64.7%)	31 (60.8%)		27 (52.9%)	36 (70.6%)	
**Revascularization strategy, no. (%)**						
None (medical therapy only)	7 (13.7%)	13 (25.5%)	0.135	11 (21.6%)	13 (25.5%)	0.641
Coronary artery bypass graft surgery	2 (3.9%)	2 (3.9%)	1.000	3 (5.9%)	2 (3.9%)	1.000
Any PCI	42 (82.4%)	36 (70.6%)	0.161	37 (72.5%)	36 (70.6%)	0.826
Balloon angioplasty only	3 (5.9%)	1 (2.0%)	0.610	3 (5.9%)	1 (2.0%)	0.610
Stent implantation	39 (76.5%)	35 (68.6%)	0.375	34 (66.7%)	35 (68.6%)	0.832
**Treated vessel(s) per patient, no. (%)**						
Left main coronary artery	1 (2.0%)	4 (7.8%)	0.359	5 (9.8%)	3 (5.9%)	0.713
Left anterior descending artery	22 (43.1%)	22 (43.1%)	1.000	19 (37.3%)	21 (41.2%)	0.685
Left circumflex artery	16 (31.4%)	7 (13.7%)	0.033	12 (23.5%)	9 (17.6%)	0.463
Right coronary artery	15 (29.4%)	16 (31.4%)	0.830	16 (31.4%)	15 (29.4%)	0.830
Ramus intermedius	0 (0.0%)	1 (2.0%)	1.000	0 (0.0%)	1 (2.0%)	1.000
≥2 vessels treated, no. (%)	11 (21.6%)	10 (19.6%)	0.807	11 (21.6%)	11 (21.6%)	1.000
Thrombus aspiration, no. (%)	0 (0.0%)	1 (2.0%)	1.000	1 (2.0%)	0 (0.0%)	1.000
Lesions treated per patient	3.98 ± 1.59	3.80 ± 1.72	0.592	4.33 ± 2.28	4.71 ± 2.17	0.399
**Lesions treated per patient**						
1	27 (52.9%)	23 (45.1%)	0.428	19 (37.3%)	24 (47.1%)	0.316
2	12 (23.5%)	8 (15.7%)	0.318	14 (27.5%)	8 (15.7%)	0.149
≥3	3 (5.9%)	5 (9.8%)	0.713	4 (7.8%)	4 (7.8%)	1.000
≥1 complex lesion (type B2/C), no. (%)	41 (80.4%)	43 (84.3%)	0.603	42 (82.4%)	40 (78.4%)	0.618
TIMI 3 flow post-procedure in all treated lesions, no. (%)	42 (82.4%)	36 (70.6%)	0.161	35 (68.6%)	35 (68.6%)	1.000
Coronary stenosis after PCI < 30% in all treated lesions, no. (%)	42 (82.4%)	35 (68.6%)	0.107	36 (70.6%)	36(70.6%)	1.000
Procedural success in all treated lesions, no. (%)	42 (82.4%)	35 (68.6%)	0.107	37 (72.5%)	36 (70.6%)	0.826
Number of stents per patient	1.39 ± 1.23	1.20 ± 1.08	0.395	1.24 ± 1.14	1.33 ± 1.41	0.700
Overall stent length per patients (mm)	37.75 ± 38.07	31.82 ± 30.45	0.388	31.14 ± 29.70	35.14 ± 39.66	0.566
Total SYNTAX score	15.27 ± 10.65	17.24 ± 11.93	0.381	19.14 ± 12.04	19.87 ± 10.67	0.745
**Medication after the operation, no. (%)**						
Aspirin	50 (98.0%)	50 (98.0%)	1.000	46 (90.2%)	46 (90.2%)	1.000
Clopidogrel	34 (66.7%)	36 (70.6%)	0.670	32 (62.7%)	38 (74.5%)	0.200
Ticagrelor	14 (27.5%)	13 (25.5%)	0.822	16 (31.4%)	9 (17.6%)	0.107
Bivalirudin	23 (45.1%)	27 (52.9%)	0.428	9 (17.6%)	9 (17.6%)	1.000
Tirofiban	5 (9.8%)	10 (19.6%)	0.162	3 (5.9%)	1 (2.0%)	0.610
Enoxaparin	23 (45.1%)	21 (41.2%)	0.689	13 (25.5%)	13 (25.5%)	1.000
Low molecular weight heparin	3 (5.9%)	2 (3.9%)	1.000	2 (3.9%)	1 (2.0%)	1.000
Rivaroxaban	0 (0.0%)	0 (0.0%)	1.000	2 (3.9%)	2 (3.9%)	1.000
Fondaparinux	1 (2.0%)	0 (0.0%)	1.000	3 (5.9%)	7 (13.7%)	0.183
**Medication at discharge, no. (%)**						
Aspirin	50 (98.0%)	50 (98.0%)	1.000	46 (90.2%)	45 (88.2%)	0.750
Clopidogrel	32 (62.7%)	33 (64.7%)	0.837	31 (60.8%)	36 (70.6%)	0.297
Ticagrelor	16 (31.4%)	16 (31.4%)	1.000	15 (29.4%)	8 (15.7%)	0.097
Angiotensin-converting enzyme inhibitors	17 (34.0%)	20 (39.2%)	0.586	4 (7.8%)	7 (13.7%)	0.338
Angiotensin II receptor blockers	15 (29.4%)	10 (19.6%)	0.250	30 (58.8%)	24 (47.1%)	0.234
Nitrate	28 (54.9%)	31 (60.8%)	0.547	35 (68.6%)	35 (68.6%)	1.000
Statins	49 (96.1%)	50 (98.0%)	1.000	50 (98.0%)	50 (98.0%)	1.000
Beta blockers	33 (64.7%)	35 (68.6%)	0.674	38 (74.5%)	35 (68.6%)	0.510
Diuretics	9 (17.6%)	7 (13.7%)	0.586	17 (33.3%)	16 (31.4%)	0.832
Insulin	4 (7.8%)	3 (5.9%)	1.000	9 (17.6%)	7 (13.7%)	0.586
Oral hypoglycemic drugs	9 (17.6%)	7 (13.7%)	0.586	7 (13.7%)	11 (21.6%)	0.299
Proton-pump inhibitor	24 (47.1%)	23 (45.1%)	0.843	23 (45.1%)	31 (60.8%)	0.113
Calcium channel blocker	12 (23.5%)	12 (23.5%)	1.000	26 (51.0%)	17 (33.3%)	0.071

*PCI, percutaneous coronary intervention; SYNTAX score, the Synergy between Percutaneous Coronary Intervention with Taxus and Cardiac Surgery score; TIMI, thrombolysis in myocardial infarction.*

**TABLE 4:** Angiographic lesion-based procedural characteristics.

Characteristics	eGFR ≥ 60 ml/min			eGFR < 60 ml/min		
	Reduced-bolus (*n* = 51)	Normal-bolus (*n* = 51)	*P*-value	Reduced-bolus (*n* = 51)	Normal-bolus (*n* = 51)	*P*-value
Number of lesions	201	194	–	221	240	–
Number of lesions with PCI, no. (%)	63	55	–	60	55	–
Lesions stented, no. (%)	60 (95.2%)	51 (92.7%)	0.853	51 (85.0%)	51 (92.7%)	0.191
Lesions not stented, no. (%)	3 (4.8%)	4 (7.3%)	0.853	9 (15.0%)	4 (11.3%)	0.191
**TIMI flow before PCI, no. (%)**						
0 or 1	19 (30.2%)	9 (16.4%)	0.142	13 (21.7%)	8 (14.5%)	0.237
2	4 (6.3%)	2 (3.6%)		4 (6.7%)	1 (1.8%)	
3	40 (63.5%)	44 (80.0%)		43 (71.7%)	46 (83.6%)	
**TIMI flow after PCI, no. (%)**						
0 or 1	0 (0.0%)	0 (0.0%)	1.000	1 (1.7%)	0 (0.0%)	0.629
2	0 (0.0%)	0 (0.0%)		1 (1.7%)	1 (1.8%)	
3	63 (100%)	55 (100%)		58 (96.7%)	54 (98.2%)	
Coronary stenosis after PCI < 30%, no. (%)	63 (100%)	54 (98.2%)	0.466	56 (93.3%)	55 (100.0%)	0.150
Procedural success, no. (%)	63 (100%)	54 (98.2%)	0.466	57 (95.0%)	55 (100.0%)	0.274
Total stent length per lesion (mm)	32.08 ± 17.64	31.82 ± 18.85	0.940	31.14 ± 16.43	35.57 ± 19.02	0.211
Average stent diameter per lesion (mm)	2.99 ± 0.44	3.00 ± 0.47	0.898	3.15 ± 0.49	2.97 ± 0.47	0.057
**Number of stents**	71	61	–	63	68	–
Pre-stenting dilation, no. (%)	69 (97.2%)	59 (96.7%)	1.000	61 (96.8%)	67 (98.5%)	0.947
Direct stenting, no. (%)	1 (1.4%)	2 (3.3%)	0.894	2 (3.2%)	0 (0.0%)	0.229
Post-stenting dilation, no. (%)	56 (78.9%)	45 (73.8%)	0.490	57 (90.5%)	62 (91.2%)	0.890
Proximal location, no. (%)	26 (36.6%)	24 (39.3%)	0.748	27 (42.9%)	21 (30.9%)	0.155

*PCI, percutaneous coronary intervention; TIMI, thrombolysis in myocardial infarction.*

All the patients included in the analyses underwent coronary angiography with the most use of radial arterial access (92.2%). PCI was performed in 74% of the patients, with 70% undergoing the stent implantation and 4% receiving the balloon angioplasty only, whereas 4% of the patients underwent CABG and 21% received the medical management. In the normal eGFR cohort, the average number of lesions treated per patient in the intervention and control group were 1.24 ± 0.97 and 1.08 ± 0.98, respectively (*P* = 0.592). In contrast, in the abnormal eGFR cohort, the average number was 1.18 ± 0.97 and 1.08 ± 1.06 in every two groups (*P* = 0.399). There was no significant difference in rates of lesions treated by stent implantation between the two groups for both cohorts (95.2% vs. 92.7%, *P* = 0.853 and 85% vs. 92.7%, *P* = 0.191). In patients with different renal functions, the baseline and postprocedural rates of TIMI flow were not significantly different in patients with reduced bolus of bivalirudin compared with the normal bolus. Medications that were prescribed at the hospital discharge are detailed in Table 3.

### Outcome

The ACT value in the reduced-bolus group was lower than that in the normal-bolus group at the different time points in the eGFR ≥ 60 ml/min cohort and eGFR < 60 ml/min cohort (Figures 2, 3 and Table 5). The ACT measured at 0, 10, and 30 min after the initial bolus of bivalirudin was comparable between the two groups for both cohorts, except for the ACT measured at 5 min (340.27 ± 59.19 vs. 366.37 ± 60.63, *P* = 0.03 and 367.43 ± 54.51 vs. 392.08 ± 43.13, *P* = 0.013, respectively, for normal eGFR cohort and abnormal eGFR cohort). The ACT in the two groups showed roughly the same trend, and the area under the curve of ACT was lower in the intervention group than in the control group (Figures 2, 3). The non-inferiority was established in the reduced bolus of bivalirudin in the intervention group compared with the normal bolus of bivalirudin in the control group in both eGFR cohorts. The one-sided 97.5% lower confidence bound for the difference in the area under the ACT curve was -0.017 and 0.0015 in eGFR ≥ 60 ml/min and eGFR < 60 ml/min cohort, respectively, both above the preset non-inferiority criterion of -0.07. There was no postoperative net adverse event and stent thrombosis before hospital discharge in each group.

**TABLE 5:** Clinical outcomes according to randomized treatment.

Characteristics	eGFR ≥ 60 ml/min			eGFR < 60 ml/min		
	Reduced-bolus (*n* = 51)	Normal-bolus (*n* = 51)	*P*-value	Reduced-bolus (*n* = 51)	Normal-bolus (*n* = 51)	*P*-value
**ACT measured**						
0 min	150.33 ± 31.86	150.71 ± 18.78	0.943	154.63 ± 17.57	150.24 ± 18.79	0.226
5 min	340.27 ± 59.19	366.37 ± 60.63	0.03	367.43 ± 54.51	392.08 ± 43.13	0.013
10 min	350.47 ± 51.64	368.47 ± 59.02	0.104	368.63 ± 55.28	369.61 ± 34.55	0.915
30 min	366.75 ± 50.28	366.98 ± 60.69	0.983	363.51 ± 45.55	361.27 ± 38.13	0.789
Maximum ACT	385.61 ± 53.24	402.57 ± 61.90	0.141	387.08 ± 56.80	400.84 ± 40.20	0.161
Area under the curve of ACT (primary endpoint)^ a^	0.55 ± 0.09	0.56 ± 0.08	<0.0001	0.55 ± 0.06	0.57 ± 0.05	<0.0001
**During the whole postoperative hospital stay** *^b^*						
**NACE**	0 (0.0%)	0 (0.0%)	1.000	0 (0.0%)	0 (0.0%)	1.000
MACCE	0 (0.0%)	0 (0.0%)	1.000	0 (0.0%)	0 (0.0%)	1.000
All cause death	0 (0.0%)	0 (0.0%)	1.000	0 (0.0%)	0 (0.0%)	1.000
Reinfarction	0 (0.0%)	0 (0.0%)	1.000	0 (0.0%)	0 (0.0%)	1.000
Stroke	0 (0.0%)	0 (0.0%)	1.000	0 (0.0%)	0 (0.0%)	1.000
Ischemia-driven target vessel revascularization	0 (0.0%)	0 (0.0%)	1.000	0 (0.0%)	0 (0.0%)	1.000
All bleeding	0 (0.0%)	0 (0.0%)	1.000	0 (0.0%)	0 (0.0%)	1.000
BARC 2–5	0 (0.0%)	0 (0.0%)	1.000	0 (0.0%)	0 (0.0%)	1.000
**Stent thrombosis**						
Definite	0 (0.0%)	0 (0.0%)	1.000	0 (0.0%)	0 (0.0%)	1.000
Probable	0 (0.0%)	0 (0.0%)	1.000	0 (0.0%)	0 (0.0%)	1.000
**6-month outcomes** *^b^*						
**NACE**	9 (17.6%)	17 (33.3%)	0.069	13 (25.5%)	9 (17.6%)	0.336
MACCE	1 (2.0%)	3 (5.9%)	0.610	8 (15.7%)	5 (9.8%)	0.373
All cause death	0 (0.0%)	0 (0.0%)	1.000	5 (9.8%)	1 (2.0%)	0.207
Reinfarction	1 (2.0%)	3 (5.9%)	0.610	2 (3.9%)	3 (5.9%)	1.000
Stroke	0 (0.0%)	0 (0.0%)	1.000	0 (0.0%)	0 (0.0%)	1.000
Ischemia-driven target vessel revascularization	0 (0.0%)	2 (3.9%)	0.475	2 (3.9%)	3 (5.9%)	1.000
All bleeding	9 (17.6%)	14 (27.5%)	0.236	5 (9.8%)	5 (9.8%)	1.000
BARC 2–5	0 (0.0%)	2 (3.9%)	0.475	2 (3.9%)	0 (0.0%)	0.475
**Stent thrombosis**						
Definite	0 (0.0%)	0 (0.0%)	1.000	0 (0.0%)	0 (0.0%)	1.000
Probable	0 (0.0%)	0 (0.0%)	1.000	0 (0.0%)	0 (0.0%)	1.000

*ACT, activated clotting time; NACEs, net adverse clinical events; MACCE, major adverse cardiac or cerebral events; BARC, Bleeding Academic Research Consortium.*

*^a^For non-inferiority; 1-side 97.5% CIs. The normal dose of bivalirudin is the reference group.*

*^b^For superiority; 2-side P-values. The χ^2^ or Fisher exact test was used for testing the differences between the 2 groups.*

### Follow-Up

**FIGURE 4:**
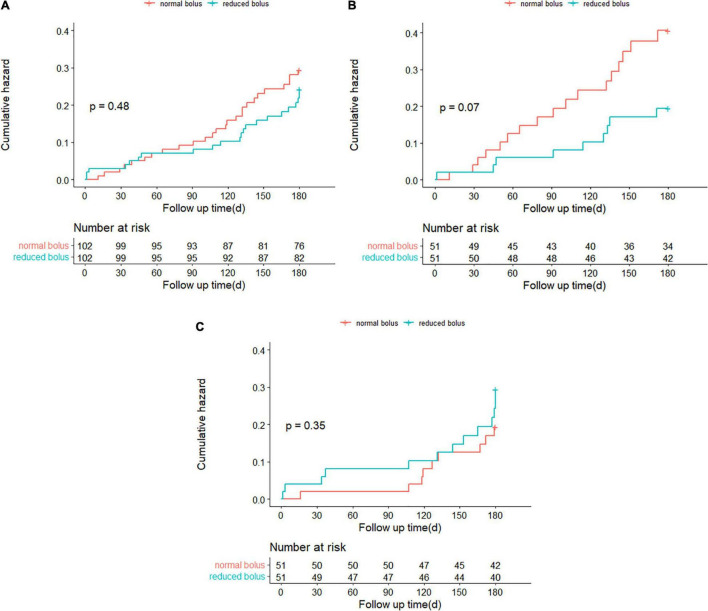
**(A)** Time-to-event curves for the net adverse clinical events (NACEs) through 6-month follow-up. **(B)** Time-to-event curves for the NACE through 6-month follow-up in patients with normal eGFR. **(C)** Time-to-event curves for the NACE through 6-month follow-up in patients with abnormal eGFR.

**FIGURE 5:**
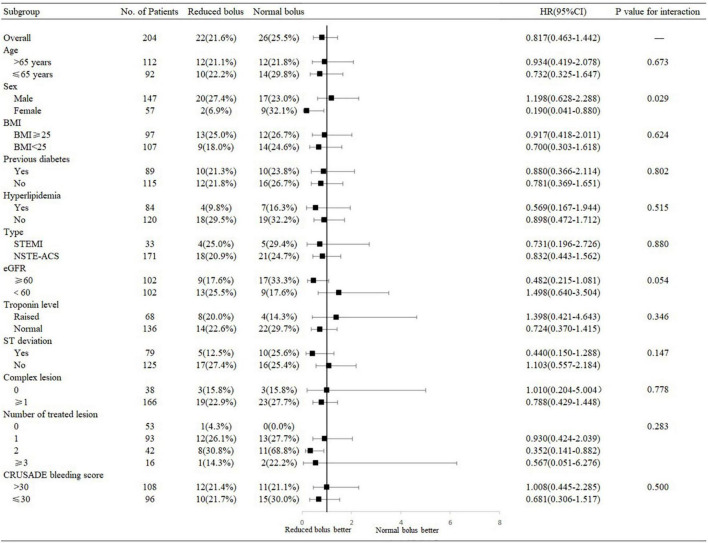
Selected subgroup analyses for the 6-month rates of NACE.

**FIGURE 6:**
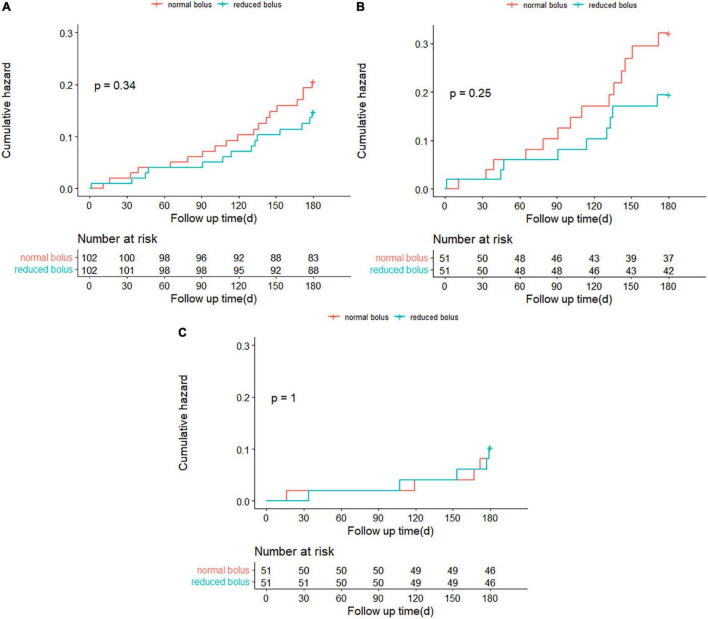
**(A)** Time-to-event curves for any Bleeding Academic Research Consortium (BARC) bleeding through 6-month follow-up. **(B)** Time-to-event curves for any BARC bleeding through 6-month follow-up in patients with normal eGFR. **(C)** Time-to-event curves for any BARC bleeding through 6-month follow-up in patients with abnormal eGFR.

Follow-up information at 6 months was complete for 204 patients, including 102 patients in the reduced-bolus group and 102 patients in the normal-bolus group (Table 5). In the reduced-bolus group, NACEs occurred in 22 patients (21.6%) in which, 9 patients had normal eGFR and 13 patients had abnormal eGFR, while in the normal-bolus group, 26 patients (25.5%) experienced an NACE including 17 patients with normal eGFR and 9 patients with abnormal eGFR [Table 5 and Figures 4A, 5; hazard ratio (HR) 0.817, 95%CI 0.463–1.442, *P* = 0.48]. A total of 14 patients (13.7%) in the reduced-bolus group as compared with 19 patients (18.6%) in the normal-bolus group had a bleeding event (Table 5, Figure 6A, and Supplementary Figure 1; HR 0.717, 95%CI 0.360–1.430, *P* = 0.34).

Among patients with normal eGFR, the rates of NACEs [9 (17.6%) vs. 17 (33.3%), HR 0.482, 95%CI 0.215–1.081, *P* = 0.07], and its individual components were not significantly different between the two groups (Table 5 and Figures 4B, 5). The proportion of individuals with any bleeding at 6 months was not significantly different in those who received the reduced bolus of bivalirudin compared with those who received normal bolus [Table 5, Figure 6B, and Supplementary Figure 1; 9 (17.6%) vs. 14 (27.5%), HR 0.614, 95%CI 0.266–1.418, *P* = 0.25]. As is shown in Table 5, the proportion of patients with MACCE was also not significantly different in those who received the reduced bolus of bivalirudin compared with those who received the normal bolus [1 (2.0%) vs. 3 (5.9%), *P* = 0.610]. No stent thrombosis at 6-month follow-up occurred in each group.

Of the patients with abnormal eGFR, 13 patients (25.5%) treated with reduced bolus of bivalirudin vs. 9 patients (17.6%) treated with normal bolus experienced an NACE event at 6-month follow-up (Table 5 and Figures 4C, 5; HR 1.498, 95%CI 0.640–3.504, *P* = 0.35). There were no statistically significant differences in the 6-month rates of any bleeding [5 (9.8%) vs. 5 (9.8%), HR 1.000, 95%CI 0.289–3.454, *P* = 1.00) nor in rates of MACCE [8 (15.7%) vs. 5 (9.8%), *P* = 0.373] in patients with reduced bolus and normal bolus of bivalirudin (Table 5, Figure 6C, and Supplementary Figure 1). There was no incidence of stent thrombosis at 6 months in each group, as is shown in Table 5. Different from patients in the normal eGFR cohort, the reduced bolus of bivalirudin resulted in numerically higher all-cause death than normal bolus in patients with abnormal eGFR [5 (9.8%) vs. 1 (2.0%), *P* = 0.207], whereas the 6-month rates of any bleeding were similar between the two groups [Table 5, Figure 6C, and Supplementary Figure 1; 5 (9.8%) vs. 5 (9.8%), HR 1.000, 95%CI 0.289–3.454, *P* = 1.00].

### Subgroup Analysis

The treatment effects of the reduced bolus of bivalirudin on the 6-month rates of the NACE and any bleeding were consistent across multiple prespecified subgroups with no significant interactions noted with regard to age, body mass index (BMI), diabetes status, hyperlipidemia, type of CHD, troponin level, ST-segment deviation, complex lesions, the number of treated lesions, and the CRUSADE bleeding score (Figure 5 and Supplementary Figure 1). The test for interaction for gender according to the intervention group was statistically significant for the NACE events (*P* = 0.029). Among male patients, the incidence of NACE was non-significantly higher in the reduced-bolus group compared with the normal-bolus group [Figure 5; 20 (27.4%) vs. 17 (23.0%), HR 1.198, 95%CI 0.628–2.288]. However, there was less NACE in the reduced-bolus group among female patients [2 (6.9%) vs. 9 (32.1%), HR 0.190, 95%CI 0.041–0.880]. There were fewer cases of bleeding with the reduced-bolus group in both male and female cases with no significant interaction (Supplementary Figure 1; *P* = 0.167). There was a non-significant trend toward an interaction effect based on the eGFR (*P* = 0.054). The incidence of 6-month NACE was higher in the reduced-bolus group than those in the normal-bolus group when eGFR < 60 ml/min [Figure 5; 13 (25.5%) vs. 9 (17.6%), HR1.498, 95%CI 0.640–3.504]. However, such events tend to be more frequent with the normal-bolus group when eGFR ≥ 60 ml/min [9 (17.6%) vs. 17 (33.3%), HR 0.482, 95%CI 0.215–1.081]. Rates of any bleeding were much the same with the reduced bolus of bivalirudin as they were with the normal bolus in patients with abnormal eGFR, but there was less bleeding in the reduced-bolus group among patients with normal eGFR [Supplementary Figure 1; 5 (9.8%) vs. 5 (9.8%), HR 1.000, 95%CI 0.289–3.454 and 9 (17.6%) vs. 14 (27.5%), HR 0.614, 95%CI 0.266–1.418 in eGFR < 60 ml/min and eGFR ≥ 60 ml/min, respectively].

## Discussion

In this prospective, randomized clinical trial involving patients undergoing cardiac catheterization with or without CKD, we found that the area under the ACT curve of reduced-dose bolus of bivalirudin was non-inferior to the area of the normal bolus. No secondary composite outcome of all-cause mortality, recurrent myocardial infarction, ischemia-driven target vessel revascularization, stroke, and bleeding events occurred after the operation but before hospital discharge in each group. After a 6-month follow-up, the reduced bolus of bivalirudin resulted in similar net clinical outcomes as did the use of normal bolus in different eGFR cohorts, which were consistent across multiple predefined subgroups. Therefore, we provided new insight into the efficacy and safety of reduced bolus of bivalirudin for patients undergoing cardiac catheterization.

ACT, a test of coagulation, is primarily used in the monitoring of UFH. The American Heart Association (AHA)/American College of Cardiology (ACC) guideline recommends that the heparin be adjusted to the ACT target range in patients receiving GPIs or not (25, 26). ACT is highly related to bleeding and ischemic complications in guiding heparin administration (27–29), whereas in guiding bivalirudin, the previous study demonstrated ACT value has not been predictive of the activity of bivalirudin (30). However, an observational study showed that ACT greater than 800 s was not associated with bleeding risk in patients receiving bivalirudin, but ACT less than 300 s could increase the thrombotic risk (31). Therefore, we chose the area under the ACT curve as the primary end point of the present trial for two main reasons. First, the peak ACT mainly used may be insufficient to reveal its correlation with the clinical events because it may be affected by the adjustment or additional infusion of bivalirudin. Second, the area under the curve of ACT could reflect the overall status of ACT, which would better balance the bleeding risk with the ischemic risk for patients undergoing cardiac catheterization. As a result, the area under the ACT curve of the reduced bolus of bivalirudin in this study was non-inferior to the normal bolus indicating the similar bleeding and ischemic risk between the two groups.

In this study, no postoperative NACEs occurred before hospital discharge. However, the incidence of NACE in bivalirudin at 6-month follow-up was higher than previously reported rates (10, 32). This finding is mostly due to the different definitions of NACE between trials. In this study, the component of the NACE that occurred more frequently was bleeding, which accounted for two-thirds of the NACE. Most of the bleeding events were mild (i.e., BARC type 1), while the major bleeding events (i.e., BARC type 2–5) were about 10%. In contrast to the previous study, we adopted the all-bleeding as the component of NACE; therefore, the incidence rate of NACE might be higher. Our study showed a non-significant reduction of NACE events in the reduced bolus of bivalirudin, which appeared to be driven by the rate of bleeding events. Among female patients, most previous studies demonstrated that the bivalirudin resulted in a non-significant lower rate of NACE compared with heparin or heparin plus GPIs (5, 7, 10, 33). Although the incidence of NACE among female patients in this study tended to be higher than the previously reported rate (10), one should be noted that reduced bolus of bivalirudin might significantly reduce the rate of NACE as compared with the normal-bolus group, which may be attributable to the efficacy of the reduced bolus of bivalirudin. The reduced bolus also insignificantly reduced the incidence of NACE in numerous subgroups examined in the same way as the normal bolus, including in high-risk patients with baseline ST-deviation, more than one complex lesion, and more treated lesions.

The low incidence of major bleeding for patients undergoing PCI was paramount since numerous studies have demonstrated the correlation between major bleeding and subsequent death (34–37). In an observational study enrolling 23,800 patients with STEMI presenting within 12 h from symptom onset, the risk of the composite of major or minor in-hospital bleeding was less in the bivalirudin group compared with the UFH group (38). In this study, no in-hospital bleeding event occurred in both two groups during hospitalization. The in-hospital bleeding risk of bivalirudin was higher in the observational study than in our study, which might be because of the high-risk population enrolled in the observational study. The magnitude of the difference might also be because of the ACT monitoring in our study. In this study, the rate of in-hospital bleeding was identical in the reduced-bolus group and the normal-bolus group, showing the efficacy of reduced bolus of bivalirudin in the clinical practice. As expected, the 6-month rate of bleeding tended to be lower with the reduced-bolus group (13.7% vs. 18.6%), although this finding was not significant. Moreover, the reduced bolus of bivalirudin resulted in a numerically lower bleeding risk compared with normal bolus among the subgroups; however, no subgroup was identified in which the bleeding risk of the patients randomly assigned to the reduced bolus of bivalirudin were significantly less than that with normal bolus.

The rate of acute stent thrombosis in this study has been shown significantly higher in the bivalirudin group than in the heparin group (5, 6, 11). However, in BRIGHT, the incidence of acute stent thrombosis in bivalirudin was identical to that in heparin or heparin plus GPIs [bivalirudin vs. heparin, RR -0.3, 95%CI -1.1–0.5) or vs. heparin plus GPIs, RR -0.1, 95%CI –0.1–0.5)] (7). In our study, there was no incidence of acute stent thrombosis in both the reduced-bolus group and normal-bolus group. The incidence of acute stent thrombosis was consistent with the low rate of acute stent thrombosis reported from the BRIGHT trial in patients receiving bivalirudin mainly because we also administered a high bolus of bivalirudin infusion at the end of the PCI, which may provide sufficient antithrombotic effect until the onset of antiplatelet effect of potent P2Y12. The low rates of definite or probable stent thrombosis in the trial at 6 months were similar to reported rates (7, 10). In our study, no difference in the incidence of stent thrombosis was identified between the reduced-bolus and normal-bolus group during the whole follow-up after PCI, which showed the reduced bolus of bivalirudin did not increase the risk of stent thrombosis even within 24 h after PCI.

In view of the high bleeding risk in patients with CKD (15–17, 39, 40), previous studies have demonstrated the beneficial effect of bivalirudin on bleeding for patients with ACS with coherent CKD compared with the heparin or heparin plus GPIs (40–42). Although the bivalirudin is an anticoagulant whose clearance is less dependent on renal function than heparin, the usage of bivalirudin in patients with renal insufficiency could also result in higher bleeding risk than those with normal renal function (4, 5, 8, 10, 43). Therefore, it is advisable to use the less guidance-recommended dosage of bivalirudin due to renal insufficiency, which will finally balance the bleeding and thrombotic risk to improve the prognosis of such patients. For patients with CKD, our study showed that no incidence of NACE, bleeding, or stent thrombosis occurred during the period of hospitalization. One would expect that the reduced bolus of bivalirudin would be better than the normal bolus in reducing the NACE and bleeding events. Conversely, our study showed a non-significant difference favoring the normal bolus of bivalirudin compared with the reduced bolus for the incidence of NACE at 6 months, although the overall risk of NACE in patients with CKD was similar to the previously reported rate (10). This result was primarily driven by the high rate of all-cause death in the reduced-bolus group, while other components of NACE were lower in the reduced-bolus group than those in the normal-bolus group. Among patients with abnormal eGFR, the reduced bolus of bivalirudin resulted in 5 cardiac deaths including 1 death caused by heart failure, while only 1 cardiac death occurred in the normal-bolus group, resulting in a trend toward better 6-month NACE of the normal bolus of bivalirudin.

To our knowledge, the REDUCE BOLUS trial is the first randomized trial to assess the efficacy and safety of reduced bolus of bivalirudin. However, several limitations of the trial should also be noted. First, patients with PCI represented a subset of 74% of all patients enrolled in our study, and those with coronary angiography only might be in a better condition, which may affect the conclusion we drew. Second, follow-up information was obtained through the review of hospital records or telephone calls, which may have resulted in bias due to the patient recall. Finally, in this study, the primary end point events were investigated during hospitalization. Although follow-up information was collected, this trial was of a relatively short duration (i.e., 6 months). Whether a longer duration of follow-up would result in significant differences between two groups in the outcome, especially in patients with CKD, remains to be elucidated.

## Conclusion

Among patients with CHD with or without CKD undergoing cardiac catheterization, no difference was observed regarding the NACEs, ischemic events, or bleeding events between the reduced bolus and normal bolus of bivalirudin suggesting the efficacy and safety of reduced bolus of bivalirudin. As a result, our findings would provide strong evidence about the usage of bivalirudin among Chinese and likely other Asian populations. Nonetheless, large studies were warranted to validate the long-term effects observed in this study.

## Data Availability Statement

The raw data supporting the conclusions of this article will be made available by the authors, without undue reservation. Further inquiries can be directed to the corresponding authors.

## Ethics Statement

The studies involving human participants were reviewed and approved by the Institutional Ethics Committee of the General Hospital of Northern Theater Command of the Chinese People’s Liberation Army. The patients/participants provided their written informed consent to participate in this study.

## Author Contributions

QH performed the data analysis and wrote the manuscript. Y-LH designed the experiments. T-NZ scrubbed the data and maintained the research data. X-ZW designed the experiments and checked the data. Q-YZ conceived and designed the experiments. All authors contributed to the article and approved the submitted version.

## Conflict of Interest

The authors declare that the research was conducted in the absence of any commercial or financial relationships that could be construed as a potential conflict of interest.

## Publisher’s Note

All claims expressed in this article are solely those of the authors and do not necessarily represent those of their affiliated organizations, or those of the publisher, the editors and the reviewers. Any product that may be evaluated in this article, or claim that may be made by its manufacturer, is not guaranteed or endorsed by the publisher.

## Supplementary Material

Supplementary Figure 1 Selected subgroup analyses for the 6-month rates of any BARC bleeding.
