# Supplementary figures and images for: Efficacy and Safety of the Reduced Bivalirudin in Patients Undergoing Coronary Angiography or Percutaneous Coronary Intervention Stratified by Renal Function (REDUCE BOLUS): A Single-Blind, Stratified Randomized, Non-inferiority Trial
Source: Front Cardiovasc Med. 2022 Apr 25;9:864048. doi: 10.3389/fcvm.2022.864048 (PMC9081606; doi:10.3389/fcvm.2022.864048)

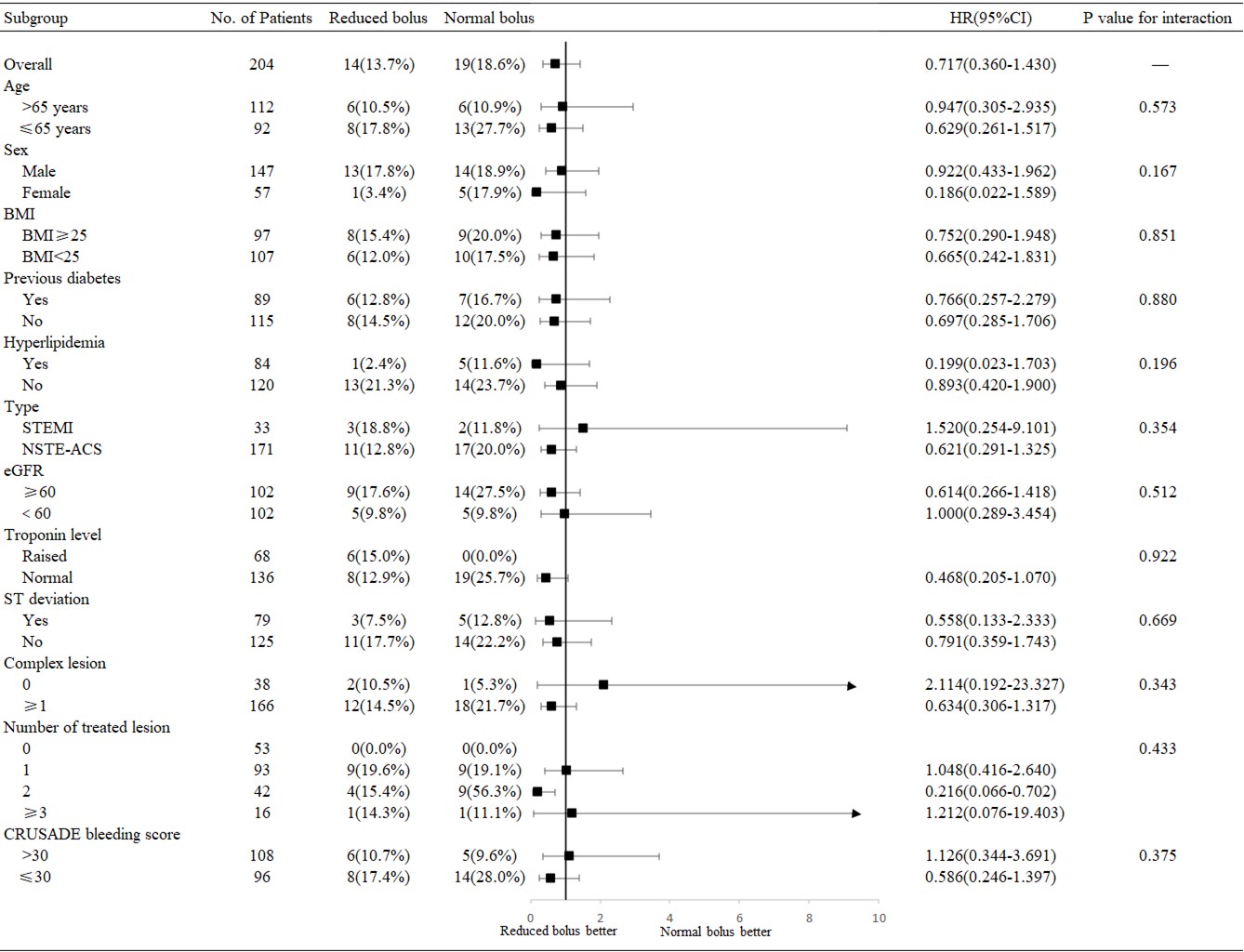

Supplement: Supplementary Figure 1 — Selected subgroup analyses for the 6-month rates of any BARC bleeding. [file Image_1.JPEG]
